# Critical perspective on infodemic and infodemic management in previous Ebola outbreaks in Uganda

**DOI:** 10.3389/fpubh.2024.1375776

**Published:** 2024-03-12

**Authors:** Sunday Jimmy Obol, Okechi Nzedibe

**Affiliations:** ^1^Freelance Consultant, Kampala, Uganda; ^2^International Public Health, Euclid University, Bangui, Central African Republic

**Keywords:** medical pluralism, health system, Ebola outbreak, structural violence, coloniality, infodemic, syndemic, exploitation and injustices

## Abstract

This research investigates the complex dynamics of Uganda’s recent Ebola outbreaks, emphasizing the interplay between disease spread, misinformation, and existing societal vulnerabilities. Highlighting poverty as a core element, it delves into how socioeconomic factors exacerbate health crises. The study scrutinizes the role of political economy, medical pluralism, health systems, and informal networks in spreading misinformation, further complicating response efforts. Through a comprehensive analysis, this study aims to shed light on the multifaceted challenges faced in combating epidemics in resource-limited settings. It calls for integrated strategies that address not only the biological aspects of the disease but also the socioeconomic and informational ecosystems that influence public health outcomes. This perspective research contributes to a better understanding of how poverty, medical pluralism, political economy, misinformation, and health emergencies intersect, offering insights for future preparedness and response initiatives.

## Introduction

The Ebola outbreak in Uganda, while successfully contained, laid bare the complex interplay between disease, misinformation, and pre-existing vulnerabilities. The World Health Organization (WHO) defines an infodemic as too much information, including false or misleading information, in digital and physical environments during a disease outbreak. In the previous Ebola outbreak, the infodemic was only realized through the physical rather than the digital environment, for reasons such as outbreaks happening in rural areas with limited access to smartphones, where literacy levels are very low, and poverty is an economic norm. Poverty, a significant factor within the affected communities, is intertwined with the infodemic fueled by social media and informal communication channels. This perspective of research examines the intersections of these forces. Poverty in Uganda manifests in limited access to healthcare, education, and reliable information. These factors created fertile ground for misinformation to flourish. Rumors about the virus’ origin, dubious cures, and government conspiracies spread rapidly, hindering containment efforts and stoking fear. Communities steeped in poverty lacked the resources and awareness to counter these narratives effectively. International donor organizations were crucial in tackling the outbreak, providing medical supplies, training healthcare workers, and supporting community engagement initiatives. However, their interventions often lacked contextual understanding, perpetuating top-down approaches that may not resonate with local cultural sensitivities or address broader structural inequalities.

## Structural violence

Infodemic is an underlying symptom and not a disease, as it is a product of the structural ambiguity of health systems, both globally and locally. Uganda’s current health system suffers from structural violence due to a lack of will by the authorities. Structural violence is defined as the “social structures-economic, political, religious, legal, and cultural that stop groups, individuals, and societies from reaching their full capability” (1). During Ebola outbreaks, humanitarianism becomes the central pillar of intervention, negating the health system’s structural challenges that should have become a priority in managing outbreaks and their ability to detect disease incidences promptly. A lack of well-equipped rural facilities to ensure surveillance, as well as the human-animal interaction embedded in culture, continues to expose the population to risk factors for the Ebola outbreak. Such a deficit has promoted health seeking and healthcare access from traditional healers. Poverty and the structures in place designed to maintain it are evident throughout all places that have had Ebola outbreaks documented. The WHO pronounced this as the world’s 25th, having been registered in settings of profound poverty (2). Ebola incident cases have never occurred in an urban setting, making it a rural panacea for those at the periphery of societal favors.

Infodemic during an Ebola outbreak is syndemic to the structural challenges that fail to detect or manage it. Syndemic is a synergistic interaction between socioecological and biological factors that result in adverse health outcomes (3). Social determinants of health, such as poverty, social inequality, social stigma, and the environment, where people live and work, have greatly affected the intensity of the syndemic. Syndemic describes how co-occurring epidemics interact biologically and occur in the sociocultural, economic, and physical environments in which they appear. The syndemic and structural violence of epidemic diseases, such as HIV/AIDS, tuberculosis, Ebola, and COVID-19, need to be understood as multilevel phenomena shaped by history, political economy, and social context (4). These provide a signal manifestation and contribution to the documentation of underlying syndemic factors; the role of power, control, oppression, and social inequality in making health and disease are abundantly evident in these studies. Unless such power imbalances are rectified in building a robust health system, syndemic conditions will always provide fertile ground for any infodemic. Structural violence remains a rural society disease, making misinformation a base for seeking care from an array of healers. This result is based on the reality of fragility and vulnerable settings where healthcare infrastructure is limited and national investments in health are inadequate.

Infodemics in the global south should not be thought of as subalterns in so far as health intervention issues in Uganda and the general global south are concerned. Instead of equitable investment in developed health systems and infrastructure that could benefit everyone, the global south is often seen as fodder for the interests of international actors, leading to uneven development and limited access to crucial resources. The continuation of inordinate mortality from Ebola in Uganda and other global southern countries is not the result of an intractable problem thwarting the global communities’ best efforts. Instead, it is a moral detachment that is subservient to the protected affluent, where mistrust has been at the backbone of achieving global health equity. Other than the lack of investment in the health system to ensure health equity, survival for the fittest remains a modern reality for many rural Ugandans when it comes to accessing quality healthcare services at all times.

## Coloniality/historical underlying mistrust

Regarding biomedicine, not everything that glitters is gold in the face of historical injustices. The establishment of health facilities followed a pattern of colonial settlement that was based on other interests instead of service provision. Colonialism deeply affected Uganda’s social fabric and inherently changed social, cultural, political, and economic structures in a way that continues to be felt to date (5). Understanding mistrust in Uganda’s population means understanding the culture of the community and the colonial impact on the establishment of social services. A history of colonialism has been a major factor in determining the health of many vulnerable population groups, and this has affected the health system and all governance systems within it. Framed as a disease control initiative, health laws were introduced in 1908 and 1909 to consolidate and later deport 33 island villages in Lake Victoria to the mainland. These regulations were a hidden method of strategically reducing the population of people from an area rich in hunting, fishing, and charcoal (6). Most times, Ebola outbreaks in Uganda have occurred in places where the population is not in support of the seating government, as sometimes the outbreak is seen as a punishment; looking at the 2001 outbreak in Gulu, as well as the 2022 Mubende and Kasanda outbreak that saw the opposition political party win the seats in the two districts.

Therefore, exposing epistemic violence by analytically ignoring the power dynamics determining levels of trust in the post-colony should be central in infodemic management. Besides, some colonial laws are still being applied, such as the Public Health Act of 1935 and the Penal Code Act of 1950. These laws sustain the deterioration of indigenous community fabrics and colonial attempts at social control (7). In the event of ensuring infodemic management, in most cases, infodemic management is seen as a flagrant display of power and disrespect toward those whose views are censored. Infodemic management should aim to demonstrate how modern social scientists should not have their moral outlooks stunted, which then delimits how they gather facts during epidemic outbreaks such as Ebola. This approach should not be through discussing counterhegemonic ways of interpreting health phenomena; instead, it should be through ways to delink knowledge production from the colonial matrix of power.

Infodemic managers, such as epidemiologists, view outbreaks through the lens of tracing the causal pathway of Ebola transmission in a “lack of trust to non-compliant actors to Ebola outbreak propagation” (8). Because of its discursive power, there is a possibility that its historical and geopolitical roots could be overlooked. Different scholars posit varied opinions on the role of infodemic managers. Richardson argues that infodemic managers prevent structural determination from becoming commonsensical by dominating how people should perceive and interpret health phenomena, therefore, such interpretations commit hermeneutic injustice (9). This means malfeasance in the way one interprets what one sees, thereby rejecting conspiracy theories as legitimate criticisms of the coloniality of power and repurposing cultural causality claims as explanations for more than a century of predatory accumulation and colonial atrocities (10). This leaves infodemic managers to ask more nuanced exploratory questions regarding misinformation and distrust during public health interventions, acknowledging structural colonial deficits and debunking them in an attempt to create more power imbalances.

Accordingly, conspiracy theories merge with other post-colonial criticisms to form truth claims that call for redistributive justice and reparations instead of bourgeois empiricism, which is characterized as gathering facts “that hide behind scientific objectivity to perpetuate dependency, exploitation, elitism, racism, and colonialism” (11). The priority of making locals understand the gravity of a public health emergency, and what is being done to arrest the situation ensures the involvement of all in managing the said epidemic. However, when infodemic managers are fronted as the crisis caravan such as the flotilla of developmental agencies and non-governmental organizations that shifts between emergencies, “scattering information aid like confetti,” (12) it exposes the social science profession as a neocolonial front for the powers that be. Therefore, there is a need for infodemic managers to pause questions before censoring any information, putting into consideration the historical and social construction of the said community. It is to help them reflect on their stance on public health emergency interventions and detach them from being crisis caravans in debunking or censoring misinformation.

The perspective of patients running away from treatment facilities is not necessarily derived from a lack of care but from historical reality. For example, the epistemic reconstitution of previous medical intervention studies aimed at eradicating human African trypanosomiasis (sleeping sickness) in French Equatorial Africa. Thirty years of archival data from French military archives show that lower levels of trust in modern medicine are correlated with greater exposure to colonial medical campaigns marked by forced lumbar punctures and treatment with aminophenyl arsonic acid (atoxyl), a somewhat effective arsenic compound that left 20% of patients blind. While this recapitulates the conflation of trust and health-seeking behavior, this is an example of how public health interventions can turn into variables for computational modeling purposes of historical and structural factors influencing how people feel about medicine and healthcare (9). In dealing with people’s fears of spreading misinformation or running away from a treatment center, explaining the current variables from the colonial ones during public health emergency interventions is very important. Information sharing should reflect empowerment through a rights-based approach. A similar intervention event created mistrust in the Belgian colony across the Congo River, where individuals suspected of having sleeping sickness were held in camps renowned for their toxic therapy, unfavorable living conditions, scarcity of food, and the permanent separation of patients from their families, all while being watched over by armed guards (9). A further good example is the research on modern mistrust associated with the awful unethical Tuskegee experiments. The lingering effects of medieval medicine serve as a reminder that mistrust does not develop in a vacuum and that “cultural” views do not supersede behaviors related to obtaining health care. Thus, as infodemic managers, our aim must not be short of past reality, but rather one that ensures knowledge is at the foundation of our duty in shaping our responsibilities.

Sociocultural knowledge has been essential to comprehending the virus and implementing containment measures in any Ebola outbreak. Social scientists on the ground have demonstrated why people reacted so negatively, even violently, to curfews and quarantines; why Ebola rumors should not be discounted as irrational or paranoid; and why grieving families chose to conceal bodies rather than turn them in for official burial (13). Countering misinformation must reflect the historical context of subjectivity. The recognition of history, politics, and culture productively liberates people from the decontextualized, faceless, and pliable role of “victim” (13). Furthermore, it becomes evident how much of the blame for the epidemic did not rest with culture itself when one examines the institutional cultures of different institutions and the government itself. Before biomedicine, a culture existed and still dominates not only health-seeking behaviors but also health and healing overall. During disease outbreaks in Uganda, despite the perception that culture was limited, irrational, or dangerous, it actually sparked specific decisions and debates inside and among a worldwide class of purported saviors.

## Health system

Infodemic management is incomplete without the role of an effective and efficient health system in the promotion of good quality health outcomes since the study of disease is characterized by the investigation of a set of factors, including biology, epidemiology, sufferer, and community understandings of the disease of concern (14), and the social, political, and economic conditions that may have contributed to the development of ill health. This is part of its effort to identify and understand health within the intersecting political economy and biosocial causality frameworks. The health system is not independent of the forces that shape its operationality, which is not limited to financing but also establishment. Therefore, the efficiency and effectiveness of the services provided will always depend on the intersecting frameworks. The vulnerability of the health system remains the sole cause of outbreaks and epidemics in Uganda. Therefore, the particularly devastating course of the Ebola epidemic’ should not be attributed to the “biological characteristics of the virus alone (15); rather, the result of the combination of “dysfunctional health systems in the country. The lack of economic independence in low-income countries, such as Uganda, has seen them fail to build robust health systems for their citizens.

The World Bank and International Monetary Fund’s conditional loans have contributed to and continued to undermine health systems in low- and middle-income countries. Besides, such negligence leads to trust issues regarding the role of health service providers, who are, in reality, incapacitated by their governing structures. Therefore, without addressing such issues, infodemic management remains more knee-jerk to underlying issues beyond a government’s means. These institutions limit public spending for Uganda and other developing countries, leading to a dependence on developmental aid funding from wealthier countries such as the United States (16). Dependence on development aid places the entire population in strict isolation as a kind of abandonment. This has deprived investment in the health system hence leading to the deaths of many Africans due to preventable epidemics or the chronicity of the different epidemics without a functioning health system.

Centuries of exploitation and injustice highlight their impact on the failings of the healthcare system. By focusing greater attention on the historical and capitalistic patterns of violence and dispossession, the need to speak to social, political, economic, and historical determinants of health and wellbeing lies at the heart of health advocacy work and approach (17). Infodemic management should not be limited to outcomes of such grounded reality; rather, they must focus on the root cause of recurrent epidemics such as Ebola to weed out the coloniality of injustices.

## Medical pluralism

There has never been a universal medical cultural practice, unless before the biomedical revolution. The different global cultures have practiced different healing practices and assigned different meanings to illness occurrences. Therefore, the practice of seeking healing has never depended on one healing approach limited to spiritual, herbal, or even biomedicine, as practiced during colonial or post-colonial times. Therefore, all these have different meanings, especially during an outbreak. The availability of various medical approaches, treatments, and institutions for individuals to utilize in their pursuit of health is medical pluralism, and it involves seeking care from several sources (18). Thus, through what prism, must we define infodemic well knowing there is no universal culture based on the reality of cultural diversity in health-seeking behaviors?

Medical practitioners and ordinary citizens are becoming more aware that we need to put into perspective cultural variations in medical belief and practice (19). Understanding how health and illness are handled in various cultural contexts helps us identify “culture-bound” aspects of our own medical practices and beliefs, as seen in the role of anthropology during different Ebola outbreaks and during the COVID-19 pandemic. Addressing misinformation requires a cultural understanding of illnesses in different cultural settings. Infodemic management is an innovation in modern industrial or post-industrial societies, and biomedicine is the dominant system. These two are factors to consider in medical pluralism. Besides, these two factors tend to exist in a competitive relationship with other systems such as chiropractic, naturopathy, Christian science, evangelical faith healing, and various folk medical systems (20). The duo is prominent based on their technological prowess, forgetting how such cannot influence cultural practices.

Understanding the confluence of biomedicine and the pharmaceutical industry, the heartbeat of biomedicine in the modern world since biomedicine has become the focus of the pharmaceutical industry. Infodemic managers should play a role in the translation of medical discoveries and package information, in addition to propagating health education. Infodemic managers also need to be anchored in understanding the cultural connotations of health and illness. They also need to understand how medical pluralism is defined by a pattern in which biomedicine exercises dominance over alternative medical systems, whether or not they are professionalized. It is this dominance that aims to leverage accurate information through censorship or gagging any unscientific information in the face of cultural diversity. When we understand how medical pluralism flourishes in all class-divided societies, it tends to mirror the wider sphere of unequal social relationships, with the patterns of hierarchy among co-present medical systems being based upon the reigning structure of class, caste, racial, ethnic, regional, religious, or gender distinctions (21). In the process of managing infodemics during recent outbreaks, there is a need to realize how it is more accurate to say that national medical systems in the modern or postmodern world tend to be plural, giving birth to different information meanings than what was structured during the colonial and pre-colonial eras. Should we claim that infodemic management will equally enjoy biomedicine dominance status over all heterodox and ethnomedical practices, knowing well how political misinformation is at the heart of modern-day infodemics?

## Conclusion

The research critically examines the multifaceted impacts of structural violence and infodemics on health outcomes, particularly in the context of Ebola outbreaks in Uganda. The research elucidates how structural violence, rooted in economic, political, and cultural systems, prevents societies from achieving their full potential, thereby exacerbating health crises. The document highlights the syndemic nature of infodemics, which, fueled by structural challenges, worsen health disparities, especially in rural settings lacking robust healthcare infrastructure. The interplay between socioecological and biological factors highlights the necessity to address social determinants of health to mitigate adverse outcomes.

Furthermore, the research delves into the historical and colonial underpinnings of mistrust in health systems, underscoring how colonial legacies continue to shape health behaviors and perceptions in Uganda. It argues for a nuanced understanding of infodemic management that acknowledges the colonial matrix of power and seeks to empower communities by contextualizing health interventions within their historical and cultural realities, looking at it from a syndemic perspective. According to (22), syndemics are “the concentration and deleterious interaction of two or more diseases or other health conditions in a population, especially as a consequence of social inequity and the unjust exercise of power.” In addressing infodemics during a disease outbreak, a syndemic framework must be used to address the biosocial relationships during outbreaks. Syndemics develop under conditions of health disparities caused by poverty, stress, and structural violence that lead to further suffering by patients whose pain could be managed but whose conditions deteriorate because of the co-occurrence of another disease.

In conclusion, this research advocates for a comprehensive approach to health crises that transcends biological interventions to include social, economic, and political considerations. It calls for the dismantling of structural violence and the coloniality of power to build more equitable and responsive health systems. Addressing the root causes of health disparities, including poverty, social inequality, and historical injustices, is essential for preventing future epidemics and ensuring that all individuals have the opportunity to achieve optimal health outcomes. The article highlights the importance of medical pluralism and cultural competency in infodemic management, emphasizing that health interventions must be grounded in the local sociocultural context to be effective.

## Data availability statement

The original contributions presented in the study are included in the article/supplementary material, further inquiries can be directed to the corresponding author.

## Ethics statement

Ethical approval was not required for the study involving humans in accordance with the local legislation and institutional requirements. Written informed consent to participate in this study was not required from the participants or the participants’ legal guardians/next of kin in accordance with the national legislation and the institutional requirements.

## Author contributions

SO: Conceptualization, Writing – original draft, Writing – review & editing. ON: Writing – review & editing.
